# A novel pH-sensitive carrier for the delivery of antitumor drugs: histidine-modified auricularia auricular polysaccharide nano-micelles

**DOI:** 10.1038/s41598-017-04428-8

**Published:** 2017-07-06

**Authors:** Yingying Wang, Pingfei Li, Fen Chen, Lianqun Jia, Qihao Xu, Xiumei Gai, Yibin Yu, Yan Di, Zhihong Zhu, Yanyao Liang, Mengqi Liu, Weisan Pan, Xinggang Yang

**Affiliations:** 1 Department of Pharmacy, Shenyang Pharmaceutical University, Shenyang, 110016 China; 2 Department of Traditional Chinese Medicine, Shenyang Pharmaceutical University, Shenyang, 110016 China; 3 Key Laboratory of Ministry of Education for TCM Viscera-State Theory and Applications, Liaoning University of Traditional Chinese Medicine, Shenyang, 110032 China; 4 Key Laboratory of Structure-Based Drugs Design & Discovery of Ministry of Education, Shenyang Pharmaceutical University, Shenyang, 110016 China

## Abstract

The study was aimed to design a novel pH-sensitive carrier to deliver antitumor drugs to increase treatment efficiency. Histidine (His)was used to modify auricularia auricular polysaccharide (AAP) by esterification. Proton nuclear magnetic resonance spectrometry was developed to characterize the His-AAP carrier and the His-AAP Paclitaxel (PTX) micelles were prepared by self-assembled organic solvent evaporation. The formation of His-AAP PTX micelles was confirmed by dynamic light-scattering, transmission electron microscopy and high performance liquid chromatography. It was found that the His-AAP PTX micelles possessed a spherical morphology with an average diameter of 157.2 nm and an 80.3% PTX encapsulation efficiency. *In vitro* release at pH 7.4, 6.5, 5.0 reached 70%, 71%, and 88%, respectively. The cell viability assay and confocal laser scanning microscope were used to evaluate the cytotoxicity and cell uptake of the His-AAP PTX micelles. Compared with Taxol, the IC_50_ of the His-AAP PTX micelles were lower after incubating for 24 h, 48 h, or 72 h (0.216 versus 0.199, 0.065 versus 0.060, and 0.023 versus 0.005, respectively). In a test of tumor-bearing mice, the His-AAP PTX micelles significantly inhibited tumor growth. These results showed that His-AAP PTX micelles are a highly promising therapeutic system for anticancer therapy.

## Introduction

Cancer is a leading cause of death worldwide and a number of strategies for cancer therapy have been investigated over the past few decades, such as radiotherapy, chemotherapy and surgical treatment. Among these, chemotherapy has become an important method for most cancer treatments because of its high efficiency compared with other treatments. Unfortunately, traditional chemical drugs have many shortcomings, such as low solubility, poor bioavailability, non-selective distribution and rapid blood clearance^1, 2^. In order to overcome these problems, nanotechnology, such as self-assembled nano-carriers^3–5^, inorganic nano-frames^6^, micelles^7, 8^ and liposomes^9, 10^ have been employed for drug delivery.

Many materials can be used as nano-carriers. Some are synthetic carriers, for example: N-alkyl-N-trimethyl chitosan derivatives^11^, poly(L-glutamic acid)-g-methoxy poly(ethylene glycol)^12, 13^, poly(ethylene oxide)-modified poly(-amino ester)^14^, folate conjugated-poly(L-lactic acid) (PLLA)-b-PEG^15^ and carbon nanotubes^16^, while some are natural products, such as chitosan^17^, 5β-cholanic acid^18^, Polysialic acid^19^, cyclodextrins^20^ and Auricularia auricular polysaccharide^21, 22^.

Auricularia auricular polysaccharide (AAP) is a kind of water-soluble polysaccharide extracted from the fruit bodies of auricularia auricular^23^. It is believed to be of high nutritional value since it has a high content of carbohydrates, amino acids, trace elements and vitamins^24^. Also, since AAP is a water-soluble natural polysaccharide it has many favorable properties, such as excellent biodegradability and biocompatibility, good safety and anticancer activity^22, 23^. However, because of its water solubility, it cannot be used alone as a drug delivery carrier. To solve this problem, in our previous work, we combined AAP with chitosan (CS) using the polyelectrolyte complexes (PEC) method to prepare AAP-CS-NPs for the delivery of Doxorubicin (DOX)^22^. In that work, we found that AAP-CS-Nanoparticles can only deliver hydrophilic drugs and release more drugs at pH7.4 than that at pH 5.0. This is important since it is known that an acidic pH is the common microenvironment in solid tumors, and the interstitial fluid in tumors has a lower pH than normal tissues (6.75 vs. 7.23), while the pH of endosomes and lysosomes is 5.0~55^25, 26^.

Because of the microenvironment of tumors, we developed a novel type of nano-micelles which was pH-sensitive.

Histidine (His) is composed of an imidazole group, a carboxyl group, and an amino group^27^ and it is regarded as being pH-sensitive because the imidazole ring has an electron lone pair on the unsaturated nitrogen that makes His amphoteric by protonation-deprotonation^28^. The pK_a_ of His is around 6.0^5, 27^ and our previous work demonstrated that AAP contains hydroxy groups^21^, while His contains a carboxy group as described above. Thus, we modified AAP with His by esterification to obtain His-AAP and, using a self-assembled method, we prepared the His-AAP PTX micelles. At neutral pH, His is expected to form a hydrophobic core of the self-assembled His-AAP micelles, meanwhile hydrophobic drugs can be encapsulated into this core. Under acidic conditions, the imidazole group of His is expected to be protonated, causing disassembly of the micelles and eventual drug release into the cytosol^29^.

Paclitaxel (PTX) has been used as a model drug for His-AAP carries. PTX, a natural diterpenoid, is a mitotic inhibitor widely used in cancer chemotherapy. It exhibits significant activity against a broad spectrum of cancers, such as breast, ovarian, lung, colon, bladder, head and neck carcinomas^30–32^. However, the problems with PTX are its low solubility in water and most pharmaceutical solvents^33^. So commercial Taxol was formulated with a high concentration of Cremophor EL and dehydrated alcohol (1:1 v/v). But, it has been found that Cremophor EL has severe adverse effects, including hypersensitivity reactions, nephrotoxicity, cardiotoxicity and neurotoxicity^34^. Also, other serious side-effects, such as hypersensitivity, fluid retention, neutropenia and nail toxicity have been observed during clinical treatments, which is mainly due to its low tumor selectivity, resulting in high toxicity to normal tissues^30^. Therefore, a novel PTX formulation strategy with a low adjuvant content, specific tumor-targeting features and a high therapeutic efficacy is required.

In this study, His was used to modify AAP by an esterification reaction, and proton nuclear magnetic resonance (^1^H NMR) spectrometry was used to characterise the His-AAP. Then, a self-assembly method was applied to obtain the His-AAP PTX micelles. Dynamic light-scattering(DLS), transmission electron microscopy (TEM), and high performance liquid chromatography (HPLC) were used to characterize the size, morphology, encapsulation efficiency and *in vitro* release of the His-AAP PTX micelles. In addition, the cell cytotoxicity and cellular uptake of the His-AAP PTX micelles were evaluated in human breast cancer cell line (MCF-7). Finally, S180-bearing mice were used to assess the antitumor efficacy of the His-AAP PTX micelles.

## Results and Discussion

### Synthesis and Characterization of His-AAP

**Figure 1:**
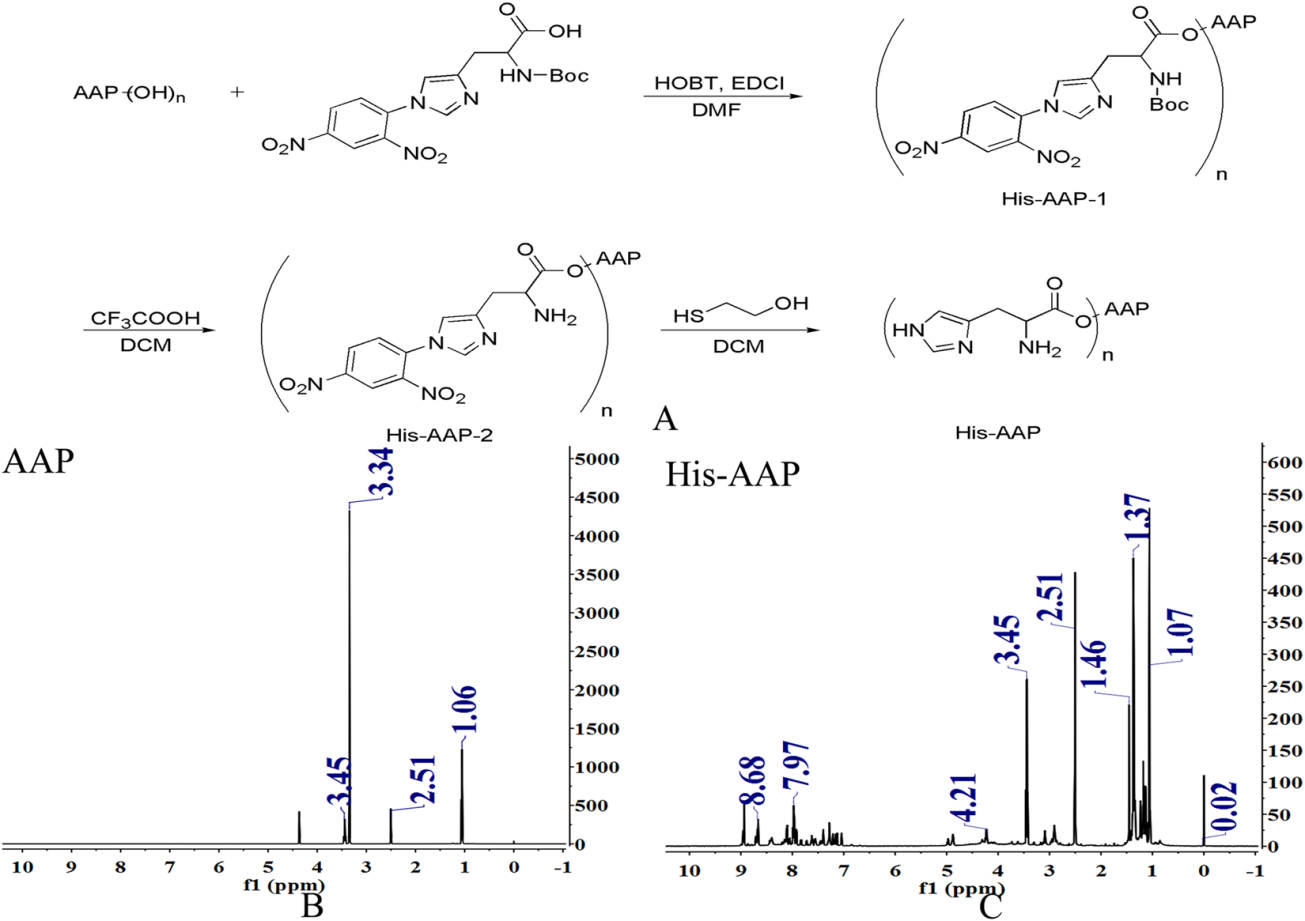
(**A**) Synthetic scheme of His-AAP. (**B**) The ^1^H NMR spectrum of AAP. (**C**) The ^1^H NMR spectrum of His-AAP.

The synthetic routes of the His-AAP were shown in Fig. 1A. The AAP and His-AAP structures were identified by proton nuclear magnetic resonance (^1^H NMR) spectroscopy (Fig. 1B and C).

As shown in Fig. 1B and C, peaks at δ (ppm) 1.06 and 1.46 were attributed to –CH_2_ and –CH. Peaks at δ (ppm) 3.34 were attributed to –CH_2_OH (Fig. 1B) while in Fig. 1C peaks at δ (ppm) 4.21 were attributed to –CH_2_-O-C=O, and the absence of peaks at δ (ppm) 3.34 indicated that we had successfully synthesized His-AAP. Peaks at δ (ppm) 7.97 were attributed to the –CH of the imidazole group (Fig. 1C).

### Characterization of His-AAP PTX micelles

**Figure 2:**
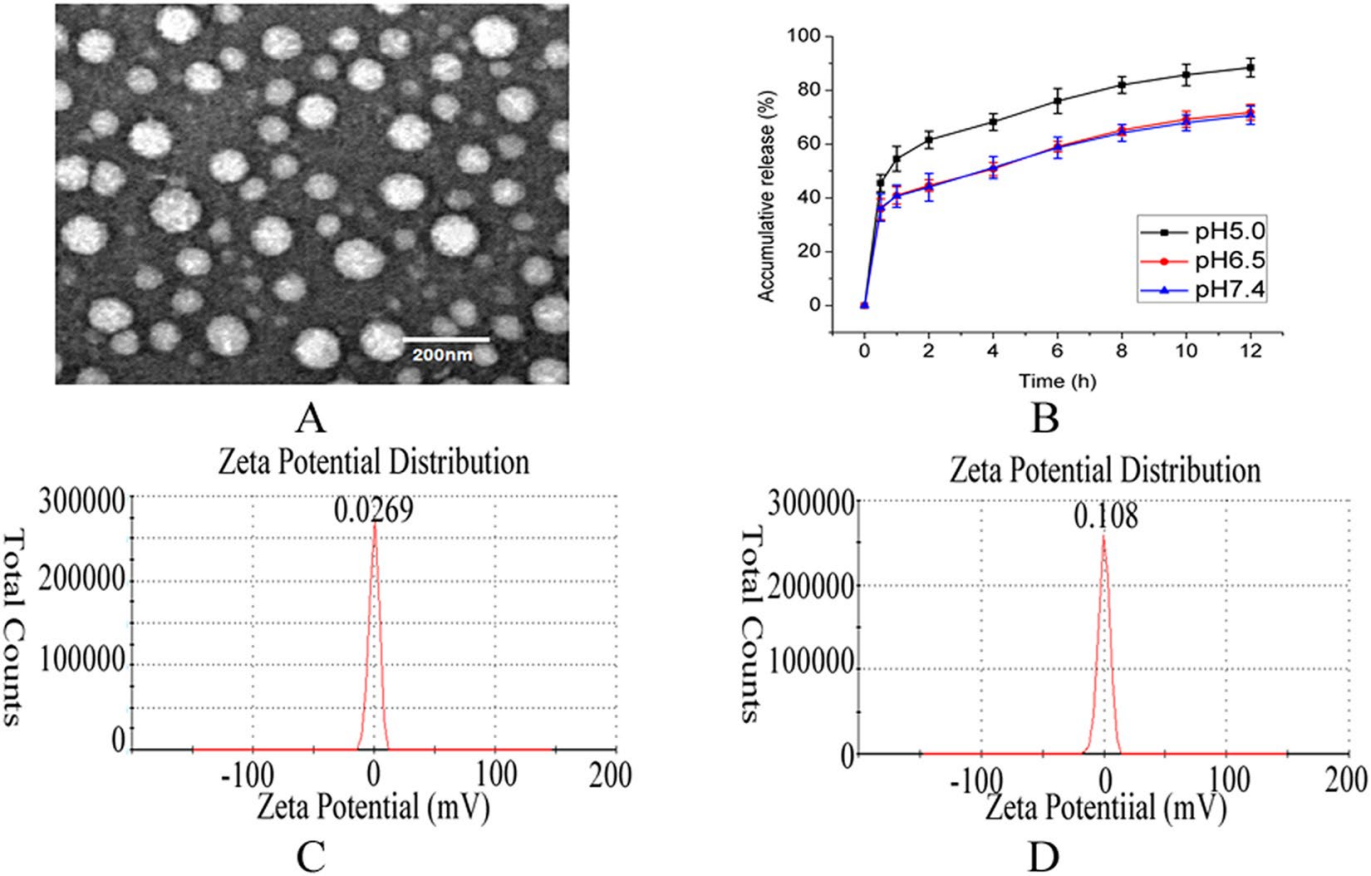
(**A**) TEM images of His-AAP PTX micelles. (**B**) *In vitro* pH-dependent PTX release profiles of His-AAP PTX micelles. (**C**) The zeta potential of blank His-AAP micelles. (**D**) The zeta potential of His-AAP PTX micelles.

A Zeta-sizer Nano instrument was used to measure the size of the micelles. As shown in Table 1, the size of His-AAP PTX micelles was bigger than blank His-AAP micelles. It should be due to the encapsulated of PTX. The particle sizes of the His-AAP PTX micelles were measured at different pH conditions. The results show that the particle sizes increased with the decreasing of pH conditions. Combined with the results of *in vitro* release, we can find that the His-AAP PTX micelles have certain pH sensitivity^27, 29^. The Polydispersity index (PDI) of samples was less than 0.5 which indicates the micelles are uniform in particle size. The zeta potential of the blank His-AAP micelles and the His-AAP PTX micelles was shown in Fig. 2C and D respectively. The encapsulation efficiency (*EE%*) and loading capacity (*LC%*) of the His-AAP PTX micelles was 80.3 ± 0.9%, 1.6 ± 1.9%, respectively.

**Table 1:** Particle sizes measured by dynamic light scattering (DLS) of micelles.

Sample	Diameter(nm)	PDI	EE(%)	LC(%)
blank His-AAP micelles	104.8 ± 3.2	0.125 ± 0.03	—	—
His-AAP PTX micelles(pH7.6)	157.2 ± 4.3	0.081 ± 0.05	80.3 ± 0.9	1.6 ± 1.9
pH7.4	179.6 ± 8.3	0.108 ± 0.04	—	—
pH6.5	185.7 ± 12.4	0.113 ± 0.03	—	—
pH5.0	378.4 ± 17.7	0.139 ± 0.05	—	—

Particle sizes measured by DLS of blank His-AAP micelles, His-AAP PTX micelles and His-AAP PTX micelles under different pH conditions after a 2 h incubation at room temperature. Data represent mean ± standard deviation, n = 3.

The TEM images of the His-AAP PTX micelles are shown in Fig. 2A. We can see that the micelles are homogeneous and spherical with a dark outer shell and a light inner core. However, the sizes of the micelles obtained by TEM were smaller than that measured by DLS. This may be due to the shrinkage of the micelles during the drying process when TEM samples were prepared. Similar observations have previously been reported^5, 35–37^.

### *In vitro* release

The PTX release rates *in vitro* from the His-AAP PTX micelles were used to evaluate the pH sensitivity of the micelles. Fig. 2B shows the cumulative release of the micelles under three pH conditions at 37 °C. The cumulative release rate at pH5.0 (~88%) is higher than that at pH6.5 (~71%) or pH7.4 (~70%). Under acidic conditions, the imidazole group of histidine is expected to be protonated, causing disassembly of the micelles and the release of PTX^27, 29^.

### Cytotoxicity assay

**Figure 3:**
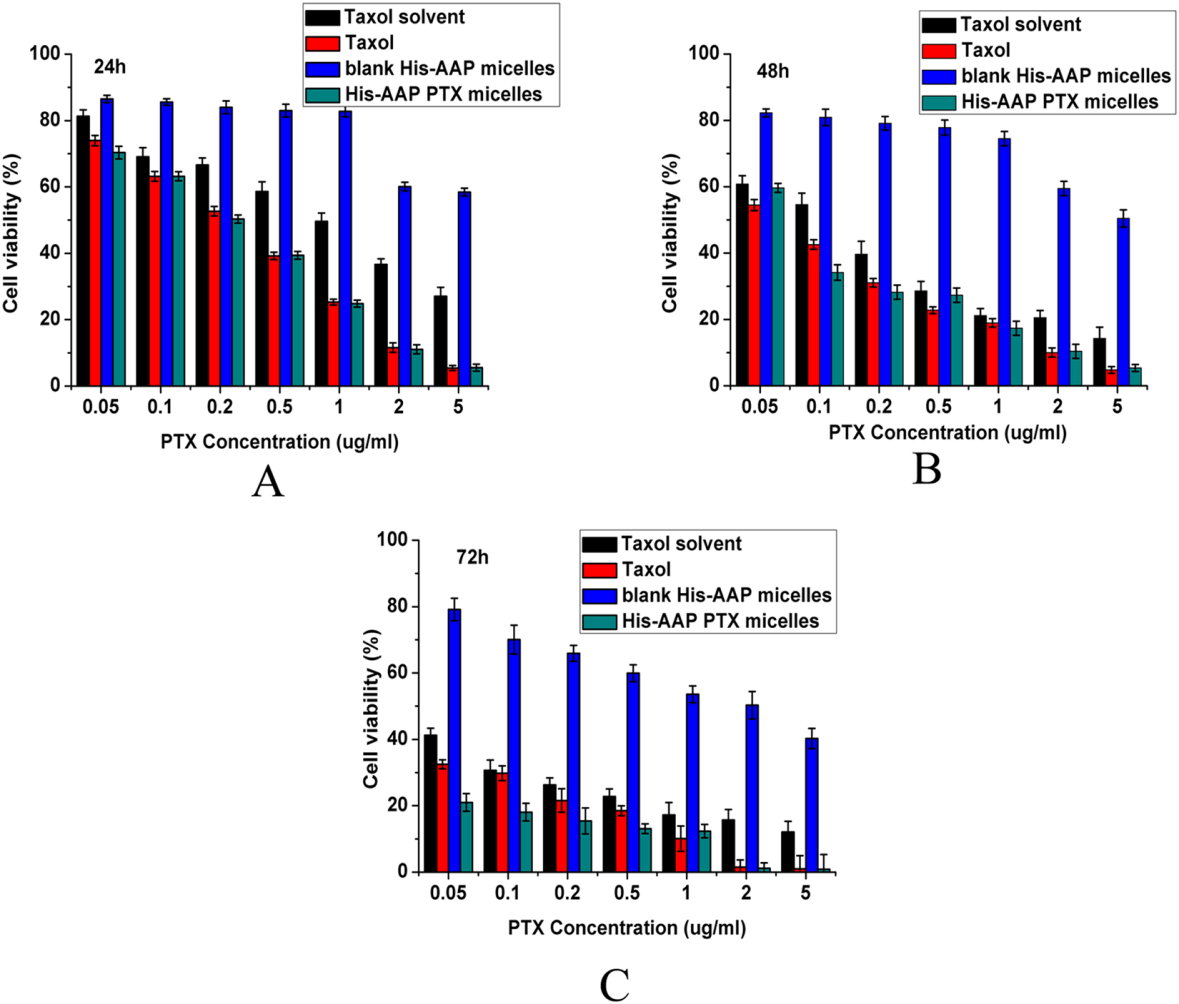
*In vitro* cell viability results determined by MTT assay of MCF-7 cells treated with Taxol solvent, Taxol, blank His-AAP micelles and His-AAP PTX micelles after 24 h (**A**), 48 h (**B**) and 72 h (**C**) incubation at 37 °C. Each well contained 4,000 cells. Data represent mean ± standard deviation (n = 3).

The antiproliferative effect of Taxol solvent, Taxol, blank His-AAP micelles, and the His-AAP PTX micelles were measured using MCF-7 cell lines for 24 h, 48 h, or 72 h. As shown in Fig. 3A,B and C, blank His-AAP micelles exhibited some cytotoxicity and with the extension of time, the cell viability decreased. This may be because of the antitumor ability of AAP^23^. The viability of MCF-7 cells decreased with an increase in the PTX concentration when the cells were cultured with Taxol or His-AAP PTX micelles for 24 h, 48 h, or 72 h. The cytotoxicity of the His-AAP PTX micelles was higher than that of Taxol. From Table 2, we can see that, compared with the Taxol group, the IC_50_ of the His-AAP PTX micelles group was lower after incubating for 24 h, 48 h, or 72 h (0.216 versus 0.199, 0.065 versus 0.060, and 0.023 versus 0.005, respectively). These results indicated the encapsulation of PTX into His-AAP micelles could increase the cytotoxicity of PTX. This may be due to the increased cellular uptake of PTX by cancer cells.

**Table 2:** Cytotoxicity of the PTX formulations against the MCF-7 cell line, expressed as the IC50 (µg/mL).

Formulations	24 h	48 h	72 h
Taxol®	0.216 ± 0.02	0.065 ± 0.005	0.023 ± 0.008
His-AAPPTX micelles	0.199 ± 0.04	0.060 ± 0.004	0.005 ± 0.001

**Figure 4:**
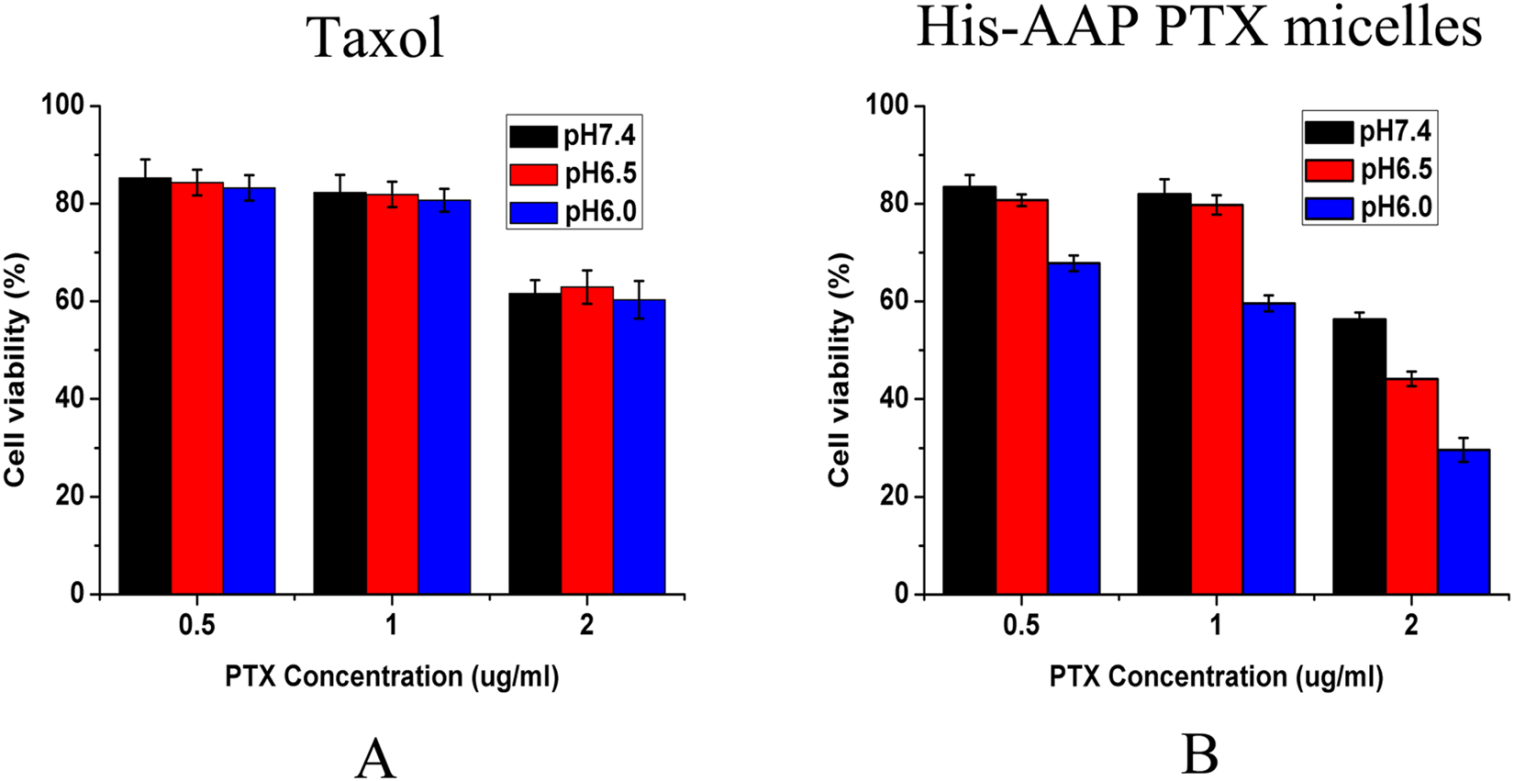
*In vitro* pH-dependent cell viability of Taxol (**A**), His-AAP PTX micelles (**B**) after 24 h incubation at 37 °C. Each well contained 4,000 cells. Data represent mean ± standard deviation (n = 3).

The cell viability of MCF-7 cell only under acidic medium was shown in supplementary Figure S1. From Figure S1, we can see the pH of medium had no influence on the cell viability. Fig. 4 shows that, along with the decline in pH, the cytotoxicity of the His-AAP PTX micelles increased, while the cell viability of Taxol changes very little. This could be because PTX was released from micelles under acidic conditions and the PTX concentration in cancer cells under acidic conditions was higher than that under neutral conditions. This phenomenon indicates that the His-AAP PTX micelles have certain pH sensitivity.

### Intracellular distribution of PTX

**Figure 5:**
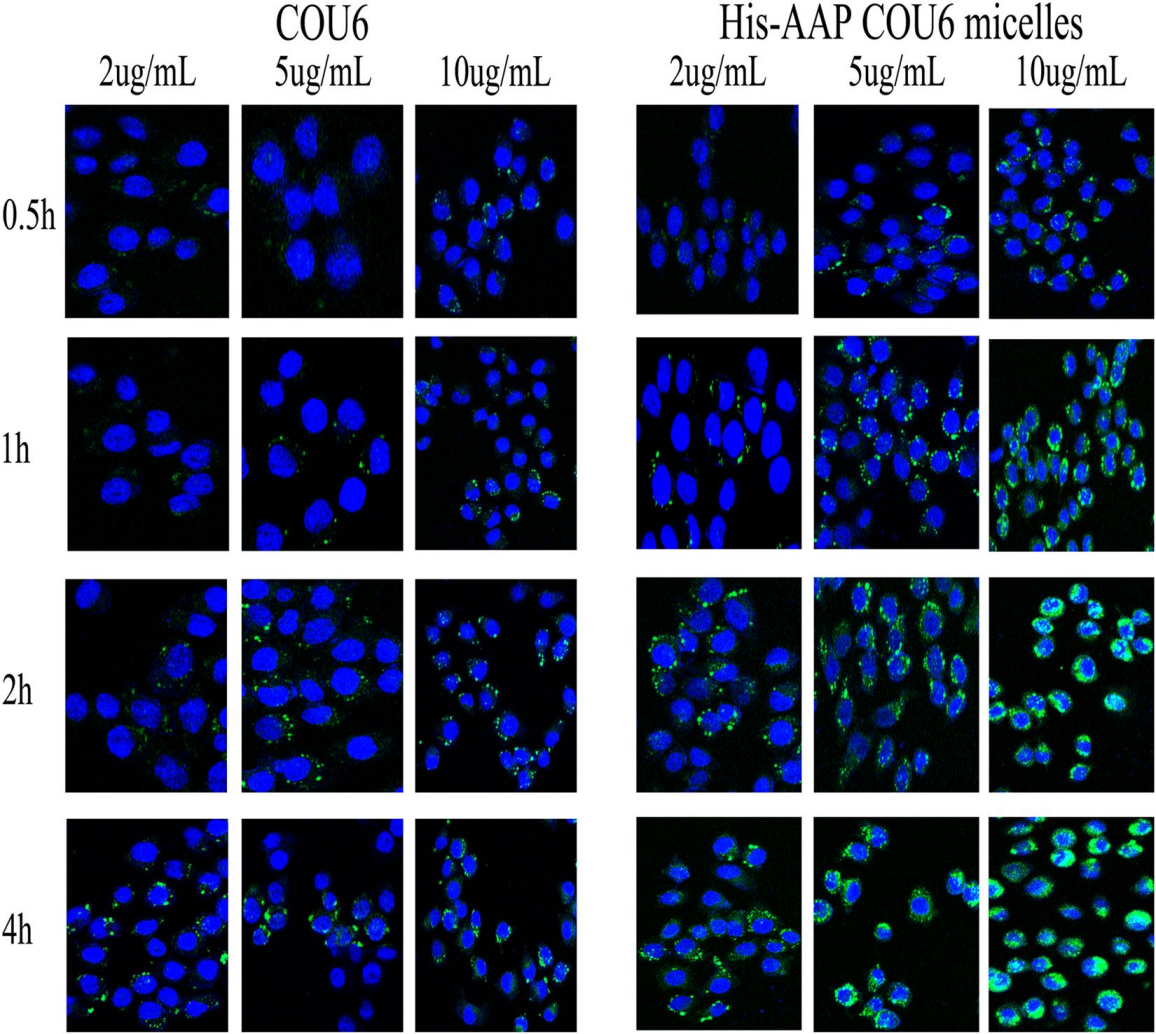
Confocal microscopy images of MCF-7 cells incubated with three different concentrations of COU6 for 0.5 h, 1 h, 2 h or 4 h, blue: fluorescence of DAPI, green: fluorescence of COU6.

Confocal laser scanning microscope (CLSM) was used to monitor the cellular uptake and intracellular distribution and the CLSM images are shown in Fig. 5. PTX and His-AAP did not produce any fluorescence for CLSM observation. Thus, coumarin 6 (COU6) was used to observe the intracellular distribution of micelles. Blue represents the cell nucleus, and green represents the color of COU6. As shown in Fig. 5, COU6-loaded micelles exhibited time-dependent in MCF-7 cells, which are first ingested into the cytoplasm, and as time goes on, the micelles enter the nucleus. At the same time, It can also be seen that the cell uptake with a concentration-dependent, the higher the concentration, the greater the cell intake. While free COU6 concentrated in the cytoplasm after 4 h incubation. This may be due to the high viscosity of AAP^38^, which causes it to tightly adhere to the cell membrane and prolongs its retention by targeted substrates, resulting in an increased drug accumulation in tumor cells. Meanwhile, the nanostructure of micelles also helps to increase the cell uptake.

### Antitumor efficacy

**Figure 6:**
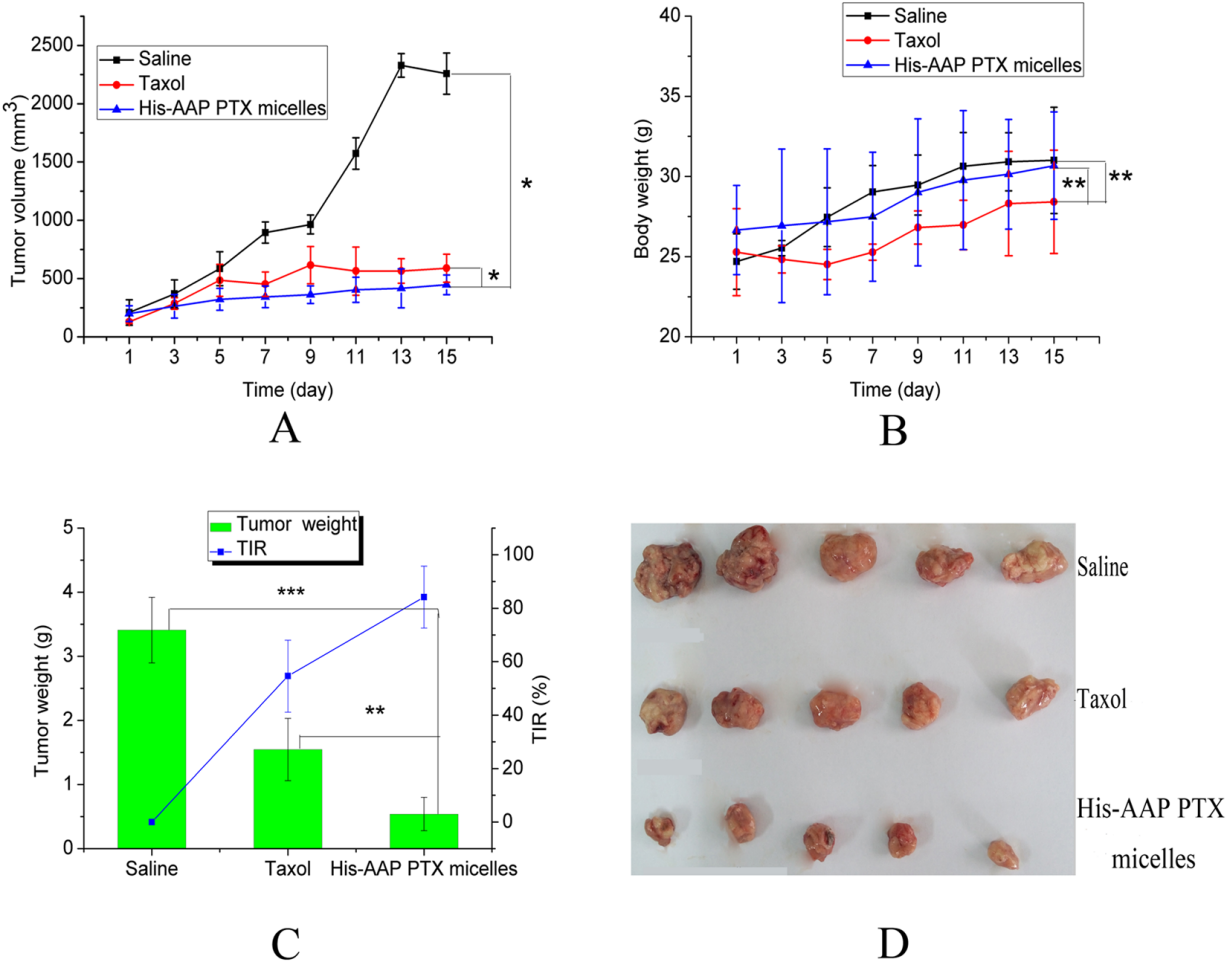
(**A**) Increase in the tumor volume of tumor-bearing mice after treatment with saline, Taxol and His-AAP PTX micelles for 15 days. (**B**) Changes in body weight of tumor-bearing mice after treatment with saline, Taxol and His-AAP PTX micelles for 15 days. (**C**) The chart shows the tumor weight after a 15-day treatment, and the line represents the TIR value. (**D**) The photograph of the excised tumors. The values are means ± SD (n = 5). Significant differences are indicated as follows: *P < 0.05, **P < 0.01 and ***P < 0.001.

The changes in tumor volume of tumor-bearing mice treated with saline, Taxol and His-AAP PTX micelles for 15 days were shown in Fig. 6A. We can see that the tumor volume in the saline group increased rapidly (from 200 mm^3^ to 2200 mm^3^), while the tumor volume of Taxol and His-AAP PTX micelles grew slowly (from 130 mm^3^ to 590 mm^3^, and from 200 mm^3^ to 440 mm^3^, respectively). The tumor growth rate of the saline group was regarded as the normal growth rate without any treatment. Compared with the saline group and the Taxol group, the His-AAP PTX micelles had a higher inhibitory effect on tumor growth (P < 0.05). The significant increase in antitumor efficacy was mainly due to the pH- sensitive nature of the micelles and the viscosity of AAP, which not only increased the concentration in the tumor but also prolonged the retention time in the tumor.

The changes in the body weight of mice treated with saline, Taxol and His-AAP PTX micelles for 15 days are shown in Fig. 6B. We can see that there was a reduction in body weight in the Taxol group, compared with the saline group and the His-AAP PTX micelles group, the Taxol group exhibited a significant reduction (P < 0.01), which may be due to the injection of Cremophor EL and the non-selective distribution of PTX.

In order to better compare the antitumor efficacy of the saline group, the Taxol group and the His-AAP PTX micelles group, the excised tumors were weighed and the tumor inhibition rate (TIR) values were calculated. As shown in Fig. 6C and D, the tumor weight of the His-AAP PTX micelles group was significantly lower than that of the saline group and the Taxol group (P < 0.001, P < 0.01,respectively). The TIR value of the His-AAP PTX micelles group was found to be 84.2%, while the TIR value of the Taxol group was 54.6%. These results indicate that the His-AAP PTX micelles group exhibited an excellent antitumor effect and lower side-effects.

## Conclusions

In this study, we successfully prepared paclitaxel-loaded pH-sensitive micelles, and confirmed that the His-AAP PTX micelles had good drug-loading and pH-sensitive *in vitro* release. Compared with commercial Taxol, the His-AAP PTX micelles exhibited higher cytotoxicity. The COU6-loaded His-AAP micelles are more likely to be ingested into cells than COU6 solutions by the experiment of Cell confocal. In the tumor-bearing mice study, the His-AAP PTX micelles significantly reduced the tumor growth, and the systemic side-effects. Therefore, the new synthetic amphiphilic, pH-sensitive copolymer His-AAP and its application in micelle formulations appears to be an effective method for the delivery of antitumor drugs and has great potential for efficient tumor therapy.

## Materials and Methods

### Materials

AAP (molecular weight 80785.1Da) was extracted from auricularia auricular^21, 39^. KM mice (female, weighting 18–25 g) free of any ocular damage were provided by the Lab Animal Center of Shenyang Pharmaceutical University (Shenyang, China). All animal studies were conducted in accordance with the Principles of Laboratory Animal Care, and approved by Shenyang Pharmaceutical University Animal Ethical Committee. The ethical committee approval number of animal studies is SYPU-IACUC-C2016-0620-104. All the other reagents and chemicals used were of analytical grade.

### Synthesis of Histidine-modified Auricularia auricular polysaccharide (His-AAP)

The general synthesis scheme of the His-AAP is shown in Fig. 1A. First, 1.0 g (2.4 mmol) histidine which was protected by t-butyloxycarbonyl (Boc) and 2, 4-dinitropheno (DNP) was dissolved in 30 mL dimethyl formamide (DMF), and the pH of the solution was adjusted to 8 with trimethylamine (Et3N). Then, 0.4 g (2.9 mmol) hydroxyl benzotriazole (HOBT) and 0.6 g (2.9 mmol) 1-ethyl-(3-dimethyllaminopropyl) carbodiie hydrochlide (EDCI) were added to the solution in an ice-bath for 30 minutes followed by the addition of 0.2 g AAP to the solution. The reaction was allowed to proceed at room temperature for 24 h. Then, the reaction mixture was extracted with ethyl acetate, and washed with saturated NaCl solution. After this, the organic horizon was combined, and dried with anhydrous MgSO_4_, suction filtrated, concentrated to obtain crude AAP1. Then the crude AAP1 was dissolved with 20 mL dichloromethane (DCM), added 5 mL CF_3_COOH to the solution for 4 h, after concentrated to obtain crude AAP2. Finally, the crude AAP2 was putted in a bottle, 2 mL mercaptoethanol was added in for 2 h, the reaction liquid was concentrated and dialyzed with a dialysis bag (MW 8000–14000). The dialysis medium was deionized water. After that, the solution was lyophilized to obtain AAP.

### Characterization of His-AAP and AAP

The chemical structures of His-AAP and AAP were characterized by ^1^H NMR spectra using D-DMSO at a concentration of 5 ~ 10 mg/mL. The ^1^H NMR spectra were recorded on a Bruker ARX-600 instrument (600 MHz, Bruker Corporation, Fällanden, Switzerland).

### Preparation of His-AAP-PTX micelles

PTX was loaded into the cores of the His-AAP micelles using self-assembly organic solvent evaporation method. Briefly, 1 mg His-AAP was mixed with a chosen amount of PTX in 1 mL methanol. The mixture solution was then added slowly to 5 mL deionized water with magnetic stirring for 1 hour at 45 °C and the nano-micelles suspension was passed through a membrane filter (0.22 µm, Millipore).

### Characterization of His-AAP-PTX micelles

#### Particle size

A Zeta-sizer Nano instrument (Malvern Instruments, UK) was used to measure the mean particle size of PTX-loaded His-AAP micelles and blank His-AAP micelles at room temperature. To measure the pH-sensitive His-AAP PTX micelles under different pH conditions, the micelles were exposed to buffers with predetermined pH values for 2 h before measurements were carried out. Each measurement was performed in triplicate.

#### TEM

The surface morphology of PTX-loaded His-AAP micelles was investigated by TEM (JEM-1200EX JEOL, Tokyo, Japan). Briefly, prepared sample solutions were dropped onto carbon-coated copper grids, then blotted, washed, negatively stained with 2% (w/v) phosphotungstic acid, air dried, and subjected to TEM.

#### Encapsulation efficiency and loading capacity

500 µL His-AAP PTX micelles were putted in ultrafiltration centrifuge tube, and centrifuged with a rapid of 5000 rpm for 10 min, repeated three times. The solutions in the EP tube were free PTX. The content of PTX was determined by HPLC using a LC-ATvp pump and SPD-10 Avp ultraviolet light detector (Shimadzu, Kyoto, Japan). HPLC conditions were as follows: a Diamasil1 C18 column (200 mm × 4.6 mm, 5 mm, Dikma, Tianjin, China) was used. The mobile phase consisted of acetonitrile and water (55:45, v/v) delivered at a flow rate of 1.0 mL min^−1^. The wavelength was set at 228 nm and the injection volume was 20 µL. The calibration curve was linear in the range of 0.1–20 mg/mL with a correlation coefficient of r^2^ = 0.9999. The weight of His –AAP PTX micelles was the plus of the weight of His-AAP carrier and PTX.

The encapsulation efficiency (*EE%*) and loading capacity (*LC%*) of the His-AAP PTX micelles were calculated using the following equation (1) and equation (2):

1 $${\rm{EE}}( \% )=\frac{{W}_{I}-{W}_{F}}{{W}_{I}}\times 100\,$$

2 $${\rm{LC}}( \% )=\frac{{W}_{I}-{W}_{F}}{{W}_{C}}\times 100$$

Where W_I_, W_F_ and W_C_ are, respectively, the weight of initially added PTX, the weight of free PTX, and the weight of His –AAP PTX micelles (unit: µg).

### *In vitro* release

The PTX release from the PTX-loaded His-AAP micelles was investigated by dialysis. For this, 1 mL PTX-loaded His-AAP micelles were transferred to a bag with a dialysis membrane (MWCO8000–14000). Then, the dialysis bag was immersed in 50 mL PBS containing 0.5% (w/v) SDS and stirred at 100 rpm and 37 °C. At predetermined time, 1 mL of external medium was withdrawn for HPLC analysis of its PTX content and replaced with the same volume of fresh medium. For the pH sensitivity studies, pH5.0, pH6.5, and pH7.4 were used for the *in vitro* release.

### Cytotoxicity assay

The cell viability was determined using MTT assay^40, 41^. The cytotoxicity of Taxol solvent, Taxol, blank His-AAP micelles and His-AAP PTX micelles was investigated using MCF-7 cells, which were maintained in RPMI 1640 medium (10% FBS; 5% CO2 at 37 °C). For this, 200 µL MCF-7 cells with a cellular density of 4.0 × 10^3^ cells/well were added to each well of a 96-well plate. After incubation for 24 h in an incubator (37 °C, 5% CO_2_), the culture medium was replaced with different PTX concentrations of Taxol solvent, Taxol, blank His-AAP micelles and His-AAP PTX micelles, diluted with RPMI1640. Then, the 96-well plate was returned to the incubator and incubated for 24 h, 48 h, and 72 h. Then, 20 µL MTT solution (5 mg/mL) was added to the wells, followed by incubation for 4 h. Finally, the culture medium was removed and 150 µL DMSO was added followed by shaking at room temperature.The viability of the cells was determined by the MTT method at 490 nm. The cell viability was calculated using the following equation (3).

3 $${\rm{Cell}}\,{\rm{viability}}( \% )=\frac{O{D}_{t}-O{D}_{b}}{O{D}_{C}-\,O{D}_{b}}$$

Where OD_t_, OD_b_, OD_c_ are, respectively, the OD of the texted group, the OD of the blank group, the OD of the control group.

In order to determine whether the micelle has pH sensitivity, we studied the cell viability of Taxol and the His-AAP PTX micelles at three different pH conditions.

Statistical Product and Service Solutions (SPSS) was used to calculate the IC50 of Taxol and His-AAP PTX micelles.

### Confocal microscopy

The cellular uptake efficiency and intracellular distribution of His-AAP micelles was evaluated by confocal microscopy. Because PTX and His-AAP have no color under confocal microscopy, so we use Coumarin-6(COU6) to replace PTX. The COU 6 is an insoluble fluorescent dye which was widely used for confocal microscopy and fluorescence microscopy^30, 42^. COU6 was encapsulated into His-AAP micelles.

For this, 500 µL cells were seeded in each well of a 24-well plate (1.0 × 10^5^cells/well) with cover slips over each well. After the cells reached 80% confluence, the culture medium was removed and free COU6, COU6-loaded His-AAP micelles were added to each well with at three different concentrations of 2 μg/mL, 5 μg/mL, 10 μg/mL for 0.5 h, 1 h, 2 h, 4 h at 37 °C. Then, the cells were washed three times with cold PBS (500 µL/well), and 4% paraformaldehyde was added to each well and fixed for 30 minutes at room temperature. Then, the cells were washed three times with cold PBS (500 µL/well), 4′, 6-diamidino-2-phenylindole (DAPI) was added for 5 minutes to stain the nuclei and the cells were washed three times with cold PBS (500 µL/well). Then, the cover slips were mounted on microscope slides using a fluorescent mounting medium. The cells were then examined and images were recorded using a confocal laser scanning microscope (CLSM, Carl Zeiss LSM 710, Germany).

### Tumor-bearing mice

Twenty-one female mice weighing 20–25 g were used to evaluate the antitumor efficacy of the His-AAP PTX micelles. Before treatment, the mice were allowed to acclimatize to their environment for five days. A sarcoma-180(S-180) cell suspension (2 × 10^6^cells in 0.2 mL saline) was injected hypodermically into the armpit of each mouse to produce the tumor-bearing mouse model. A slide caliper was used to measure the length of the longest tumor axis (L, mm) and the shortest axis(S, mm), and the tumor volume was calculated using the following equation (4).

4 $${\rm{V}}=\frac{L\times {S}^{2}}{2}$$

When the tumor volume reached about 100 mm^3^, the tumor-bearing mice were divided into three groups of five mice each experiment. The mice then received an intravenous injection of saline, Taxol and His-AAP PTX micelles at day 1, 4, 7, 10 and 13 at a dose of 3 mg/kg. The volumes of the tumor and the body weight of each mouse were measured every two days after the first administration. Then, after a 15-day treatment, the mice were euthanized, and the tumors were excised and weighed. The tumor inhibition rate (TIR) was calculated using the following equation (5).

5 $${\rm{TIR}}( \% )=\frac{{W}_{s}-{W}_{t}}{{W}_{S}}\times 100$$

Where W_t_ was the tumor weight of the test group and W_s_ was the tumor weight of the saline group.

### Ethical Approval

KM mice (female, weighting 18–25 g) free of any ocular damage were provided by the Lab Animal Center of Shenyang Pharmaceutical University (Shenyang, China). All animal studies were conducted in accordance with the Principles of Laboratory Animal Care, and approved by Shenyang Pharmaceutical University Animal Ethical Committee. The ethical committee approval number of animal studies is SYPU-IACUC-C2016-0620-104.

## Electronic supplementary material

Supplementary infromation
