# Supplementary material for: A novel pH-sensitive carrier for the delivery of antitumor drugs: histidine-modified auricularia auricular polysaccharide nano-micelles
Source: Sci Rep. 2017 Jul 6;7:4751. doi: 10.1038/s41598-017-04428-8 (PMC5500601; doi:10.1038/s41598-017-04428-8)
Supplement: Supplementary file 1 — Supplementary infromation [file 41598_2017_4428_MOESM1_ESM.pdf]

A NOVEL PH-SENSITIVE CARRIER for the DELIVERY of ANTITUMOR DRUGS: HISTIDINE-MODIFIED AURICULARIA AURICULAR POLYSACCHARIDE NANO-MICELLES

Yingying Wang[1], Pingfei Li[2], Fen Chen[3], Lianqun Jia[3], Qihao Xu[4], Xiumei Gai[1], Yibin Yu[1], Yan Di[1], Zhihong Zhu[1], Yanyao Liang[1], Mengqi Liu[1], Weisan Pan[1], Xinggang Yang[1,*]

[1]Department of Pharmacy, Shenyang Pharmaceutical University, Shenyang, 110016, China

[2]Department of Traditional Chinese Medicine, Shenyang Pharmaceutical University, Shenyang, 110016, China

[3]Key Laboratory of Ministry of Education for TCM Viscera-State Theory and Applications, Liaoning University of Traditional Chinese Medicine, Shenyang, 110032, China

[4]Key Laboratory of Structure-Based Drugs Design & Discovery of Ministry of Education, Shenyang Pharmaceutical University, Shenyang, 110016, China

[*]Corresponding author:

E-mail: yangxg123@163.com (Xinggang Yang)

Tel Number: 86-24-43520532

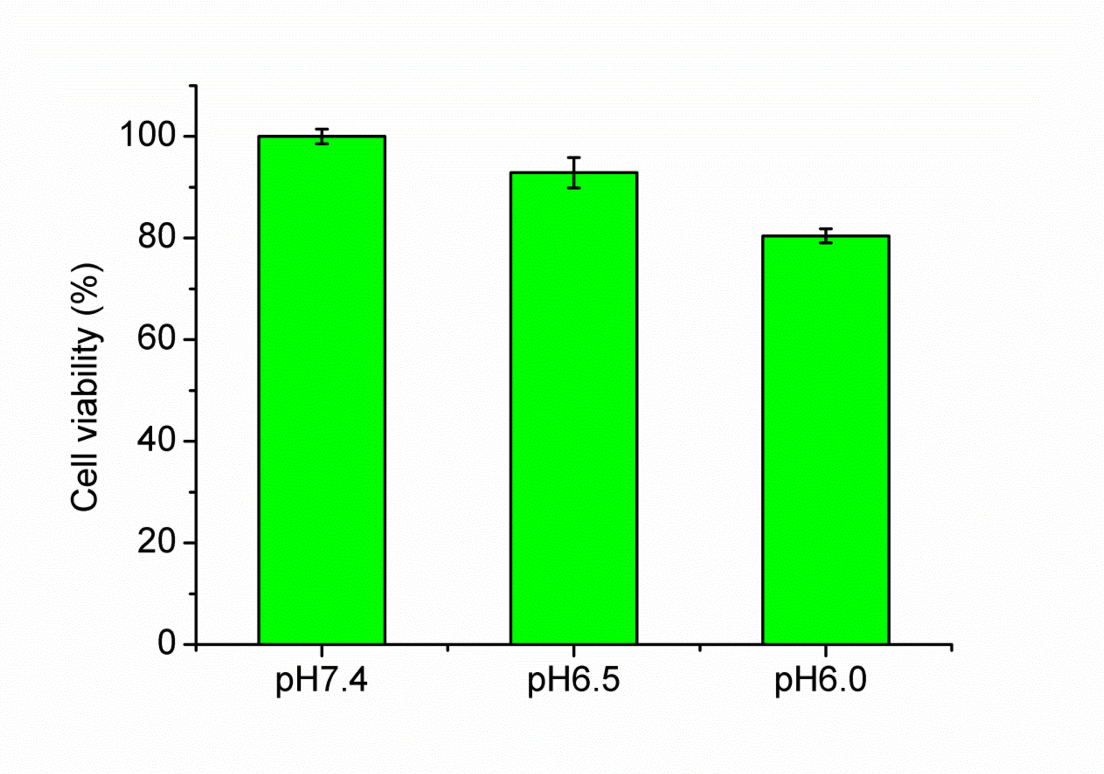

Figure S1. The cell viability of MCF-7 cell under acidic medium for 24h
